# Copper-streptocycline application modulates pomegranate (*Punica granatum* L.) secondary metabolism and antioxidant pathways against *Xanthomonas axonopodis* pv. *punicae-*induced bacterial blight

**DOI:** 10.3389/fpls.2025.1661602

**Published:** 2025-09-15

**Authors:** Ghulam Mustafa, Ishtiaq A. Rajwana, Hafiz Nazar Faried, Syed Atif Hasan Naqvi, Uzman Khalil, Muhammad Umer Iqbal, Mysoon M. Al-Ansari, Mohamed S. Elshikh, Hakim Manghwar, Fen Liu

**Affiliations:** ^1^ Lushan Botanical Garden, Jiangxi Province and Chinese Academy of Sciences, Jiujiang, Jiangxi, China; ^2^ Department of Horticulture, Muhammad Nawaz Shareef University of Agriculture, Multan, Pakistan; ^3^ Department of Plant Pathology, Faculty of Agricultural Sciences and Technology, Bahauddin Zakariya University, Multan, Pakistan; ^4^ Horticultural Sciences Department, University of Florida, Gainesville, FL, United States; ^5^ Department of Plant Pathology, Muhammad Nawaz Shareef University of Agriculture, Multan, Pakistan; ^6^ Department of Botany and Microbiology, College of Science, King Saud University, Riyadh, Saudi Arabia

**Keywords:** pomegranate, bacterial blight, *Xanthomonas axonopodis* pv. *punicae*, cultivar resistance, copper oxychloride, streptocycline

## Abstract

**Introduction:**

Bacterial blight of pomegranate (*Punica granatum* L.), caused by *Xanthomonas axonopodis* pv. *punicae* (Xap), is a major constraint to pomegranate production and fruit quality. Effective management strategies are essential to mitigate yield losses and orchard decline.

**Methods:**

Field experiments were conducted over three consecutive seasons to evaluate the efficacy of six bactericides applied at 15-day intervals (April–July) on three pomegranate cultivars such that, Sindhuri, Kalehar, and Sava using a randomized complete block design. Treatments included copper oxychloride (3000 ppm) combined with streptocycline (500 ppm) (T7) alongside other bactericides. Disease incidence, severity, marketable yield, fruit weight, aril quality, and biochemical parameters were recorded and statistically analyzed.

**Results:**

The T7 treatment proved most effective, significantly reducing bacterial blight intensity. In the Sindhuri cultivar, mean disease incidence was lowest on leaves (3.51%), fruits (3.88%), twigs (0.58%), and trees (3.59%). Disease severity values were similarly minimized, with the highest mean disease reduction observed on leaves (77.63%), fruits (79.76%), twigs (76.10%), and whole trees (76.90%). T7 also improved productivity, with marketable yield (89.60 kg), fruit weight (245 g), and hundred-aril weight (43.30 g), while enhancing biochemical traits such as TSS (17.90 °Brix), vitamin C (36.50 mg/100 mL), antioxidants (86.40% inhibition), and enzymatic activities (CAT: 21.30 U/mg protein; POD: 1.35 U/mg protein).

**Discussion:**

Among the cultivars, Sindhuri displayed the highest resistance, followed by Kalehar and Sava. The copper-streptocycline combination not only suppressed bacterial blight but also enhanced fruit yield, quality, and biochemical composition. These findings demonstrate the potential of optimized bactericide application in sustaining pomegranate productivity and reducing orchard losses due to *Xap*.

## Introduction

Pomegranate (*Punica granatum* L.) belongs to the family *Lythraceae* and has emerged as a marvelous fruit globally (Özcan et al., 2019). Pomegranate cultivation began in 4000 BC and has been successfully cultivated in Afghanistan, China, Iran, Iraq, Israel, India, Pakistan, South Africa, Spain, and the USA (Kahramanoglu et al., 2019; Singh et al., 2020). Pomegranate is considered an elixir of life and is declared a superfruit because of its color, refreshing juicy arils, potential antioxidants, and high medicinal and nutritional value (Arora et al., 2016). A global production of approximately 3.8 million tons has been reported, and the cultivated area for this crop is continuously increasing due to high consumer demand (Kahramanoglu et al., 2019). In Pakistan, the total cultivated area of pomegranate declined from 14,200 hectares (2003-2004) to 5,200 hectares (2020-2021), representing a 63.4% reduction. Production decreased from 0.0621 million tons to 0.042 million tons during the same period (32.4% reduction) (PBS, 2022; FAO, 2023). Despite pomegranate’s reputation as a hardy crop (tolerant of drought, poor soils, and high temperatures), growers face significant challenges in sustainable production. Key constraints include inadequate pruning practices, sunscald, fruit cracking, fruit flies, fungal diseases, and bacterial blight (*Xanthomonas axonopodis* pv. *punicae*) (Melgarejo et al., 2000).

Bacterial blight of pomegranate was first observed in 1988 at Chakwal “32.9328° N, 72.8630° E” and Islamabad “33.6995° N, 73.0363° E” in Pakistan (Akhtar and Bhatti, 1992). This disease was considered a minor issue before the 1990s, but it has now emerged as a primary constraint for pomegranate production. The causal agent *Xanthomonas axonopodis* pv. *punicae* (*XaP*) has been declared a quarantine pest in various pomegranate growing areas of the world, especially in Pakistan, India, South Africa, and Turkey. This can cause serious blight disease outbreaks affecting commercial cultivation and hampering the pomegranate industry (Icoz et al., 2014; Sharma et al., 2017). *XaP* is a gram-negative, monotrichous, rod-shaped, and non-sporing bacterium (Sharma et al., 2017). *Xap* infection begins with water-soaked lesions on the abaxial side of leaves in irregular patterns, with 2.50 mm to 5.00 mm in diameter (Kumar et al., 2011; Arora, 2014). The symptoms primarily appear on the leaves and fruits, followed by chlorosis and necrotic brown to black-brown spots with dried silvery bacterial ooze, approximately 15 days after infection (Sharma et al., 2017; Maity et al., 2018). The necrotic lesion on the fruit increases with the increase in fruit size, leading to “L”, “Y”, or “Star” shaped cracks appearing on the fruit surface (Mondal and Mani, 2012).

*XaP* can easily survive on fallen leaves from December to mid-March. It reproduces from mid-March to the end of June as the temperature and humidity levels become favorable for multiplication (Sharma et al., 2011). This pathogen infects through wounds and stomatal openings, causing water-soaked lesions that develop into irregular spots. It spreads via airborne cells, survives in the soil for four months, and causes fresh infections on new growth (Sharma et al., 2011). Kale et al. (2012) reported that in Punjab, the pathogen survived in infected fallen leaves for up to 210 days and in canker lesions for up to 80 days. The epidemiology of *Xanthomonas axonopodis* pv. *punicae* (*Xap*) exhibits distinct seasonal patterns: while the pathogen remains viable year-round across broad temperature ranges (9-43 °C) at lower humidity (Jadhav and Sharma, 2011), disease severity intensifies during rainy seasons under high humidity (>80%) and moderate temperatures (25-35°C). Under these favorable conditions, *Xap* proliferates within lesions on leaves, stems, and fruits. Bacterial ooze emerges from lesions during wet periods and disseminates via wind-driven rain, facilitating secondary infection of new growth (Lalithya et al., 2017).

Exposure to sunlight rapidly deactivates *Xap* in soil and on plant surfaces, leading to rapid drying and death within a few days in the soil and a few months in the plant excretophore buried in the soil. However, *Xap* can persist for years in infected plant tissues if they are kept dry and free from soil contents (Yenjerappa et al., 2004, 2006; Sharma et al., 2017; Maity et al., 2018). The increased emergence trend is attributed to various primary sources, including infected planting material, insect vectors, humans, rain splashes during thunderstorms, farm equipment, and irrigation water (Sharma et al., 2013). The pathogen (*XaP*) enters the plant body through natural openings (stomata, hydathodes, and lenticels) and wounds made by its thorns, insects, and pruning tools (Maity et al., 2018). If proper management is not followed during the fruit development and maturation stages, the chance of disease development increases manyfold and may cause up to 100% fruit loss (Singh et al., 2015). To address this devastating disease and its impact on yield and quality, we evaluated the efficacy of six bactericides, including an optimized copper-streptocycline combination, under field conditions across three consecutive seasons.

Currently, various cultural, chemical, and biological approaches are commonly used to manage bacterial blight in pomegranate orchards. Cultural practices include the removal and destruction of infected plant material, proper pruning to improve air circulation and sunlight penetration, and avoidance of overhead irrigation to prevent bacterial spread in healthy trees. Cultural control, followed by chemical control, has proven to be effective in managing bacterial blight in pomegranates. This involves pruning diseased twigs and branches, followed by four sprays of copper oxychloride + streptocycline or copper hydroxide + streptocycline, applied at 15-day intervals from mid-June to late July. These mixtures help to reduce disease indices on fruits and leaves, likely due to their ability to reduce inoculum and fresh infections (Kumar et al., 2018). Biological control involves the use of beneficial microorganisms or natural compounds to suppress pathogenic bacteria (Chen et al., 2023; Naqvi et al., 2025b). Biopesticides containing beneficial bacteria or fungi can compete with pathogens or directly inhibit their growth (Naqvi et al., 2025a). Breeding programs aim to develop pomegranate cultivars resistant or tolerant to specific bacterial diseases, offering sustainable long-term control, though requiring time and investment. Regular orchard monitoring for signs of bacterial diseases and other abiotic issues, particularly nutritional imbalances, early detection and intervention, prevention of outbreaks, and reduction of the need for intensive control measures (Kumar et al., 2018).

The effective management and complete eradication of this disease have become a challenge for researchers because of its faster inoculum build-up rate, rapid spread, and long-term survival rate in soil and planting material (Mondal and Mani, 2012). Cultural control, followed by chemical control, has been shown to be effective in managing bacterial blight infections. Application of bactericides, such as copper oxychloride and streptocycline, could help to improve yield, weight, juice (%), peel thickness, peel content, and various biochemical attributes (Shokrollah et al., 2011; Hussain et al., 2017, 2019). In Pakistan, especially in South Punjab, growers have been facing a serious threat of bacterial blight for the last 15 to 20 years.

Therefore, considering the potential significance of bacterial blight disease in pomegranate, this study aimed to evaluate the effect of various bactericides against bacterial blight disease incidence and severity and their impact on different physical, physiological, and biochemical attributes of the pomegranate fruit, so that recommendations may be disseminated to the main stakeholders nationally and internationally.

## Materials and methods

### Study site and plant selection

The current study was carried out at two distantly located pomegranate orchards, i.e., Qasba Marral (29.9812° N, 71.4240° E Multan) and Ala Bad (28°56′N, 70°58′E, Rahim Yar Khan) in South Punjab (30.0174**°** N, 71.398° E Pakistan during 2018-2019, 2019-2020, and 2020-2021. *Xap* seems to infect all available pomegranate cultivars in Pakistan, and no resistant or tolerant cultivars have been reported to date. The three most cultivated cultivars in South Punjab, Kalehar, Sindhuri, and Sava, were selected for this experiment. Eight-year-old plants with homogenous vigor and size were randomly selected for the evaluation of various bactericides against bacterial blight (**Table 1**).

**Table 1:** List of antibacterial compounds evaluated against bacterial blight disease of pomegranate.

Treatment	Trade name	Active ingredient	Active ingredient %* with formulation	Mode of action	Dose/Liter of water (ppm)	Manufacturer
T0	Water (Control)	H_2_O	H_2_O	H_2_O	H_2_O	H_2_O
T1	Cobox	Copper oxychloride	50% (WP)	Contact	3000	Swat Agro Chemicals (Pvt.) Ltd.
T2	Kocide 3000	Copper hydroxide	52.40% (WG)	Contact	2500	FMC Corporation
T3	Streptomycin	Streptomycin sulphate	75%(WG)	Contact+Systemic	500	Abbott Laboratories
T4	Streptocycline	Streptomycin sulphate + Tetracycline Hydrochloride	75%(WG)	Contact+Systemic	500	Abbott Laboratories
T5	Fornax	Bismerthiazole	20% (WP)	Contact+Systemic	2000ppm	FMC Corporation
T6	Hokko Kasomin	Kasugamycin	02% (SL)	Systemic	500ml	Arysta LifeScience
T7	Cobox+Streptocycline	Copper oxychloride + Streptocycline	50% (WP) + 75% (WG)	Contact+Systemic	3000 + 500	Swat Agro Chemicals (Pvt.) Ltd. + Abbott Laboratories

WP, Wettable powder; WG, Wettable granules; SL, Soluble liquid; *Active ingredient % with formulation” refers to the concentration of the active chemical ingredient within the commercial formulation.

### Antibacterial compound preparation and application

Antibacterial compound preparation and application followed a standardized protocol. Aqueous solutions of each treatment (concentrations detailed in **Table 1**) were freshly prepared by dissolving compounds in distilled water to a final volume of 3 L per replicate, followed by vigorous stirring for 5 minutes to ensure homogeneity. Prior to foliar application, infected plant material was surgically removed, and pruning wounds were coated with Bordeaux paste/mixture to eliminate inoculum reservoirs. Treatments were applied at 15-day intervals during the active growing season (April–July) using a calibrated high-density polyethylene (HDPE) knapsack sprayer (16 L capacity). Foliar spraying targeted the entire canopy, including abaxial leaf surfaces, until runoff was achieved (≈500 mL solution per tree). Bordeaux applications were reapplied to pruning sites after each surgical intervention to maintain protective coverage.

### Disease assessment

The experiments were conducted in the field with a Randomized Complete Block Design (RCBD), comprising a total of 40 leaves per replication/plant and ten leaves from each side. Similarly, 16 twigs and 16 fruits (four twigs and four fruits randomly selected from each side of the plant) were tagged for further assessment. The data was recorded at a regular interval of 15 days regarding disease severity, disease incidence, and disease reduction on leaves, fruits, twigs, and overall trees (Wheeler, 1969). The assessment of bacterial infestations was observed following a disease rating scale (0 to 5) where every digit represents a different infestation level, that is 0= healthy, 1= <1% infection, 2 = 1-10% infestation, 3 = 11- 20% damage, 4 = 21-50% and 5= >50% infection (Naz et al., 2018). The disease incidence, severity, % reduction over control, and disease severity of trees were calculated using the following equations.


$$Disease\;Severity\;(\%)=\;\frac{Sum\;of\;all\;the\;score\;of\;Individual\;sample\times 100}{Total\;Samples\;observed\times Maximum\;scale}$$

$$Disease\;incidence\;(\%)=\frac{Sample\;with\;BB\;symptoms}{Total\;number\;of\;Samples}\times 100$$

$$Disease\;reduction\;over\;control\;(\%)=\;\frac{DI\;in\;control-DI\;in\;treatment\;}{DI\;in\;control}\times 100$$

### Estimation of physical and biochemical quality attributes

Marketable yield (%) was calculated using a digital weighing balance (Sanyo Digital Balance). At the same time, the weight (g) of individual fruits and the hundred arils weight were measured using an electronic weight balance (OHASU Corporation, USA). The organoleptic or palatability rating of the fruits was analyzed by a panel of ten judges (seven men and three women) aged between 20 and 35 years. The committee considered the characteristics of fruit, i.e., the appearance, taste, texture, and eating quality for their evaluation. The nine-point Hedonic scale (Amerine et al., 1965) was used to evaluate sensory attributes of pomegranate fruits, including taste, texture, aroma, and overall acceptability. The total soluble solids (TSS) of aril juice were determined using a digital refractometer (PAL-1, ATAGO, Japan), and expressed in °Brix, and titratable acidity (TA) was determined using an automatic titrator (Hannan) and expressed in % citric acid. The pH of the juice was measured using a calibrated Milwaukee MW804 pH meter.

### Estimation of vitamin C, total phenolic contents, and total antioxidant activity

#### Vitamin C

Vitamin C (ascorbic acid) (mg/100ml) was estimated using the iodine titration method, which is based on the principle that vitamin C reduces iodine (I_2_) to iodide ions (I^−^) while being oxidized to dehydroascorbic acid. The assay was performed using a known concentration of vitamin C solution, which was prepared by accurately weighing 0.250 g of pure ascorbic acid, dissolving it in distilled water, and making up the volume to 250 mL in a volumetric flask. This standard solution (approximately 1 mg/mL) was used to standardize the iodine solution. During standardization, 25 mL of the standard vitamin C solution was taken in an Erlenmeyer flask, and about 10 drops of 0.5% starch indicator were added near the endpoint of titration. The iodine solution was then titrated until a faint blue-black color persisted for at least 20 seconds, and the volume of iodine used was recorded. For the sample analysis, 25 mL of the filtered sample solution was titrated similarly with the standardized iodine solution in the presence of starch indicator until the endpoint was reached (Hussain et al., 2017). Vitamin C concentration in the sample is calculated using the formula:


Vitamin C (mg/100 mL) = (V × N × 88 × 100) / v

Where V is the volume of iodine solution used for the sample (mL), N is the normality of the iodine solution, 88 represents the molecular weight of ascorbic acid, and v is the volume of the sample taken (mL). This concentration may be adjusted if the sample was diluted before titration.

#### Total phenolic contents

Total phenolic content (TPC) was determined using the Folin–Ciocalteu colorimetric assay. An appropriate amount, 0.5 mL of the diluted sample extract was added to 2.5 mL of 10% (v/v) Folin-Ciocalteu reagent, followed by the addition of 2 mL of 7.5% (w/v) sodium carbonate solution. The reaction mixture was then incubated at room temperature in the dark for 30 minutes to allow for color development. The absorbance of the resulting, blue-colored complex was measured at 765 nm using a UV-Visible spectrophotometer. A calibration curve was prepared under identical conditions using gallic acid standard solutions at known concentrations. The total phenolic content in the samples was calculated from the gallic acid standard curve and expressed as milligrams of gallic acid equivalents (mg GAE) per 100 g of the sample. All measurements were performed in triplicate, and the results were presented as the mean ± standard deviation (Razzaq et al., 2013).


$$Total\;phenolic\;contents\;(\frac{mg\;GAE}{100\;g})=\;\frac{C\;\times V\;\times D\;\times 100}{W}$$

Where C is the Concentration of gallic acid determined from the calibration curve (mg/mL), V = Volume of the extract used for the assay (mL), D = Dilution factor of the sample, W = Weight of the sample extracted (g).

#### *Antioxidant* scavenging activity

The antioxidant scavenging activity of the samples was determined using the 1,1-diphenyl-2-picrylhydrazyl (DPPH) radical scavenging assay, following the method described by Razzaq et al. (2013) with slight modifications. A freshly prepared 0.1 mM DPPH solution in methanol was used as the radical source. Briefly, 1.0 mL of the sample extract at various concentrations was mixed with 1.0 mL of DPPH solution in a test tube. The mixture was then vortexed thoroughly and incubated in the dark at room temperature for 30 minutes to allow the reaction to occur. After incubation, the absorbance of the resulting solution was measured at 517 nm using a UV-visible spectrophotometer (Cecil-CE7400S, UK) against a methanol blank. A control containing methanol and DPPH solution without sample extract was also prepared (Zheng et al., 2007). The percentage of DPPH radical scavenging activity (percent inhibition) was calculated using the following formula: A_0_.


$$\%Inhibition=\frac{(Ao\;-As)\;\;}{Ao}\times 100$$

Where *A*_0_ = absorbance of the control (DPPH solution without sample), *As* = absorbance of the sample with DPPH solution, and the assay was performed in triplicate, and the results were expressed as mean ± standard deviation. This method quantifies the ability of the extract to donate hydrogen atoms or electrons to neutralize the stable DPPH free radical, reflected by a decrease in absorbance. The method complements antioxidant activity measurements obtained from the Folin-Ciocalteu assay conducted at 765 nm.

### Determination of superoxide dismutase, catalase, and peroxidase

Sixteen fruits were harvested from each treatment, and five fruits were randomly selected for biochemical analysis. The arils were mixed, and 300 g of arils were randomly picked and stored at –80 °C. One gram of frozen sample was homogenized with 2 ml of phosphate buffer (pH 7.2) in a mortar and pestle to determine enzyme activity. The homogenized mixture was centrifuged at 12000 rpm at 4 °C for 4 min, and the supernatant was collected separately. The antioxidative enzymes, including SOD, CAT, and POD, were determined by the method of (Jin et al., 2023), with some modification using Epoch ELISA Reader (Bio-Tek Instruments, Inc., Vermont, USA).

#### Superoxide dismutase activity assay

Superoxide dismutase (SOD) activity was quantified spectrophotometrically by its ability to inhibit the photochemical reduction of nitroblue tetrazolium (NBT), a method adapted from standard protocols. The assay principle relies on superoxide radicals, generated by the photoreduction of riboflavin, reducing NBT to produce a blue formazan dye. SOD, if present, competes with NBT for these superoxide radicals, thereby inhibiting NBT reduction and consequently reducing the color intensity. The activity is thus inversely proportional to the color formed. For each assay, a total reaction volume of 3 mL was prepared. The reaction mixture contained 0.1 mM EDTA, 130 mM L-methionine, 0.75 mM NBT, and 50 mM phosphate buffer (pH 7.8). To this mixture, 0.1 mL of the prepared enzyme extract supernatant from the pomegranate samples was added. The reaction was initiated by the addition of 0.02 mM freshly prepared riboflavin. Immediately following riboflavin addition, the tubes were exposed to a 40-watt fluorescent lamp for precisely 10 minutes. Concurrently, a series of controls and blanks were prepared: a control tube (illuminated, containing all reagents but without enzyme extract) representing 100% NBT reduction; a blank tube (non-illuminated, containing all reagents but without enzyme extract) kept in the dark to determine background absorbance; and a sample blank (non-illuminated, containing all reagents and enzyme extract) also kept in the dark to account for any intrinsic absorbance of the enzyme extract. After the 10-minute illumination period, the absorbance of all reaction mixtures, controls, and blanks was measured at 560 nm using a UV-visible spectrophotometer (e.g., UV Vis 3000, Bio-Rad Inc., Hercules, California, USA). One unit (U) of SOD activity was defined as the amount of enzyme required to achieve 50% inhibition of NBT photoreduction (Jin et al., 2023). The percentage inhibition of NBT reduction for each sample was calculated using the following formula:


SOD activity=(Abs. of blank−Abs. of tested sample)÷(Abs. of blank)÷50%×(Total quantity of reaction)÷(Conc. of protein in enzyme extract)

#### Catalase activity assay

Catalase (CAT) activity was assayed spectrophotometrically by measuring the rate of decomposition of hydrogen peroxide (H_2_O_2_), following a modified version of the method described by previous studies. The reaction mixture consisted of 50 mM potassium phosphate buffer (pH 7.0), 12 mM H_2_O_2_, and 50 µL of enzyme extract, brought to a final volume suitable for the measurement. The assay was initiated by adding the enzyme extract to the substrate mixture, and the decrease in absorbance of H_2_O_2_ was monitored continuously at 240 nm over a period of 3 minutes using a UV-visible spectrophotometer (e.g., Cecil CE7400S, UK) maintained at 25 °C. The decomposition of H_2_O_2_ by catalase causes a decline in absorbance, which is proportional to enzyme activity. One unit of catalase activity (U) was defined as the amount of enzyme that catalyzes the decomposition of 1 µmol of H_2_O_2_ per minute under the assay conditions (Jin et al., 2023). An extinction coefficient of 43.6 M^-1^ cm^-1^ was used to calculate the catalase activity in µmol H_2_O_2_ decomposed min^-1^ mg^-1^ protein (U mg^-1^ protein), according to the formula:


$$CAT\;activity=\frac{Total\;volume\;in\;cuvette\times \Delta A240\;min^{-1}}{The\;Volume\;of\;enzyme\;extract\times Protein\;conc.\times 43.6\;M^{-1}\;cm^{-1}}$$

#### Peroxidase activity assay

Peroxidase (POD) activity was determined spectrophotometrically by monitoring the increase in absorbance at 470 nm, which indicates the formation of tetraguaiacol, an oxidation product of guaiacol. This method was adapted from established protocols. The reaction mixture consisted of 33 mM potassium phosphate buffer (pH 6.1), 16 mM guaiacol, 2 mM H_2_O_2_, and 200 µL of the enzyme extract. The reaction was initiated by the addition of the enzyme extract, and the increase in absorbance at 470 nm was continuously monitored for 3 minutes using a UV-visible spectrophotometer. A control reaction (without enzyme extract) was also monitored to account for any non-enzymatic oxidation. One unit (U) of POD activity was defined as the amount of enzyme that consumes 1 µmol of H_2_O_2_ per minute under the assay conditions (Jin et al., 2023). POD activity was calculated using an extinction coefficient of 26.6 mM^-1^ cm^-1^ for tetraguaiacol, and the results were expressed as µmol H_2_O_2_ decomposed min^-1^ mg^-1^ protein (U mg^-1^ protein) according to the formula. All measurements were performed in triplicate.


$$POD\;activity=\;\frac{Total\;volume\;in\;cuvette\times \Delta A470\;min^{-1}}{The\;Volume\;of\;enzyme\;extract\times Protein\;conc.\times 26.6\;M^{-1}\;cm^{-1}}$$

### Statistical analysis

All collected datasets were analyzed using a two-factor factorial analysis of variance (ANOVA) for a Randomized Complete Block Design (RCBD) with four replicates. The factors were Bactericide Treatment (8 levels: T0-T7) and Cultivar (3 levels: Kalehar, Sindhuri, Sava). Analyses were performed using OriginPro 2024 software (OriginLab Corporation, Northampton, Massachusetts, USA), following established statistical principles (Steel et al., 1997). Mean separation was conducted using Fisher’s Least Significant Difference (LSD) test at a significance level of α = 0.05.

## Results

### *Xanthomonas axonopodis* pv. *punicae* infection symptoms in pomegranate

**Figure 1:**
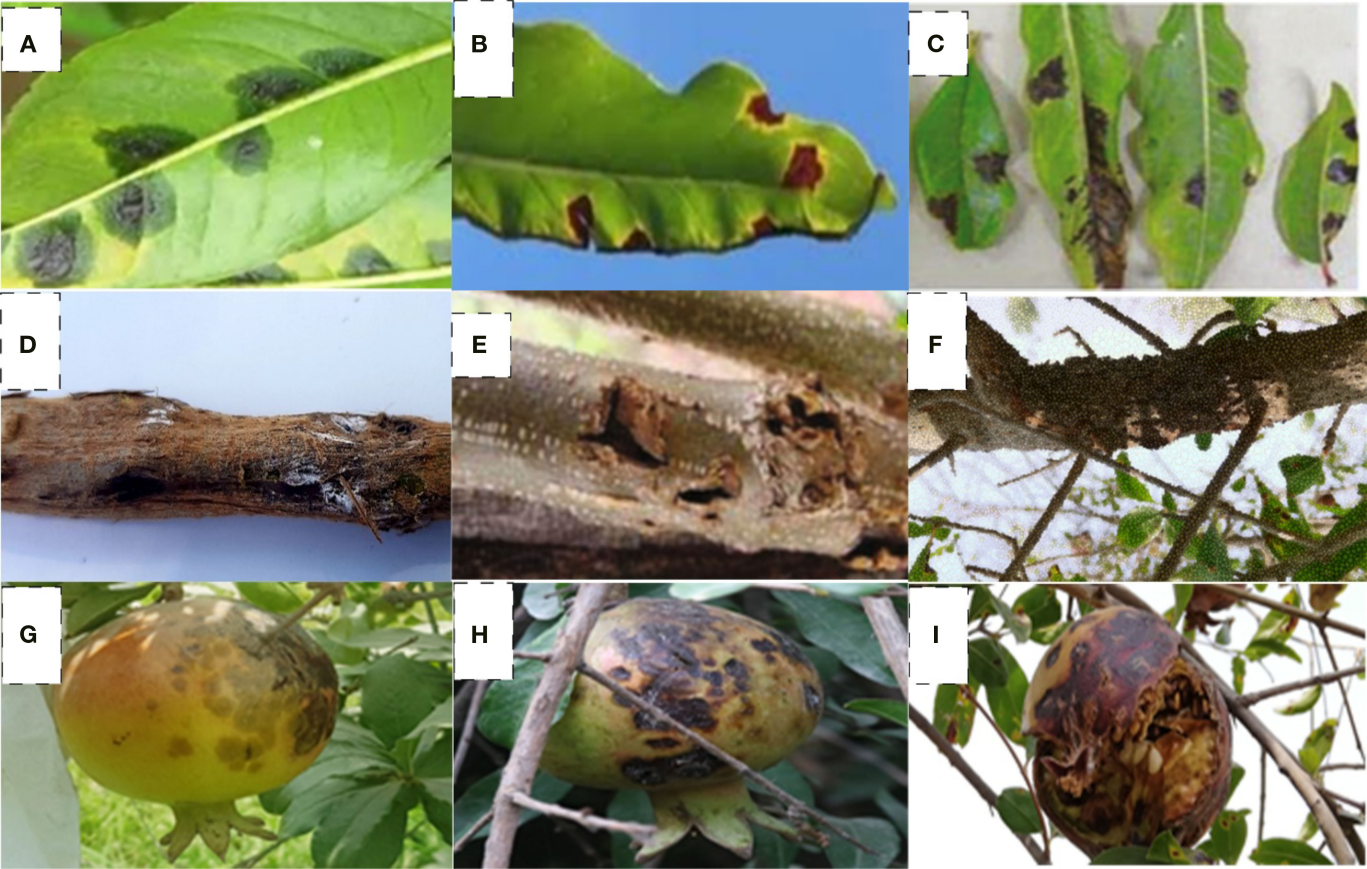
Progression of *Xanthomonas axonopodis* pv. *punicae* infection symptoms in pomegranate. **(a–c)** Leaf manifestations: initial water-soaked lesions **(a)**, angular leaf spots **(b)**, and severe bacterial ooze-induced necrosis **(c)**, **(d–f)** Woody tissue symptoms:- twig lesions **(d)**, branch cankers **(e)**, and advanced branch dieback **(f)**, **(g–i)** Fruit pathology:- coalescing water-soaked spots **(g)**, persistent pathogen colonization **(h)**, and terminal fruit rot **(i)**.

The cultivars evaluated, such as Sindhuri, Kalehar, and Sava, represent regionally dominant commercial varieties in South Asian pomegranate orchards. Crucially, all three were found universally susceptible to *Xanthomonas axonopodis* pv. *punicae* (*Xap*) with no documented genetic resistance, establishing them as standardized susceptibility benchmarks under field conditions. However, within this context of shared vulnerability, our results reveal significant treatment-mediated differential resilience. Sindhuri consistently demonstrated superior physiological responsiveness to copper-streptocycline application, exhibiting markedly lower disease penetration and enhanced activation of antioxidant pathways compared to Kalehar and Sava despite equivalent pathogen pressure. Field studies revealed characteristic bacterial blight symptoms on all parts of trees, wherein leaves exhibited water-soaked lesions progressing to necrotic spots, fruits developed coalescing lesions with internal discoloration, and woody tissues showed cankering and dieback (**Figure 1**).

### Effect of bactericides on disease development

#### Disease incidence

**Figure 2:**
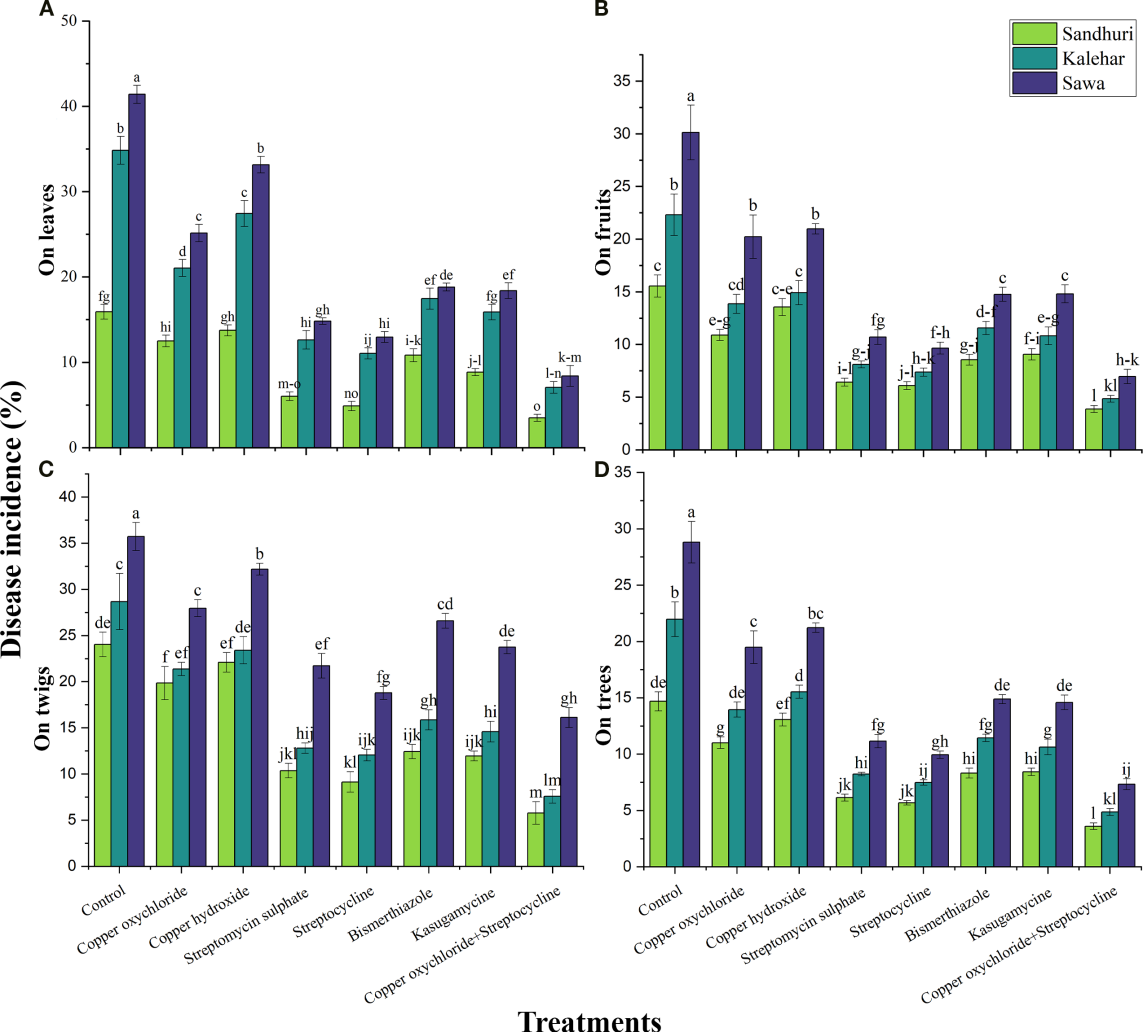
Effect of various antibacterial compounds against bacterial blight incidence (%) on Leaves **(A)**, Fruits **(B)**, Twigs **(C)**, and Tree **(D)** on three cultivars of pomegranate. The vertical bars indicate ± standard errors (S.E) of means. Means followed by the same letter in each column are not significantly different at *P ≤ 0.05*. LSD value for disease incidence on leaves= 2.55, disease incidence on fruits= 2.82, disease incidence on stem= 2.05, and disease incidence on tree= 0.34.

The interactive effect of bactericides and pomegranate cultivars on disease incidence on leaves, fruits, twigs, and trees was found to be statistically significant (*P ≤ 0.05*). The lowest disease incidence on leaves (3.51%), fruit (3.88%), twigs (0.58%), and trees (3.59%) was recorded in Sindhuri when a mixture of copper oxychloride and streptocycline (T7) was applied. Regardless of cultivar, T7 significantly reduced disease incidence by 5.11, 3.95, 3.02, and 4.06-fold on leaves, fruits, twigs, and trees, respectively, as compared to the control. In Sindhuri, the disease incidence on leaves and fruits was 1.94 to 2.31 and 1.2 to 1.7-fold less than in Kalehar and Sava, respectively. The disease incidence on twigs was reduced to 1.17-1.78-fold of that in Kalehar and Sava cultivars. At the same time, on trees it was 1.77-fold and 1.38-fold of that in Sava and Kalehar, respectively, regardless of treatment effects (**Figure 2**).

#### Disease severity

**Figure 3:**
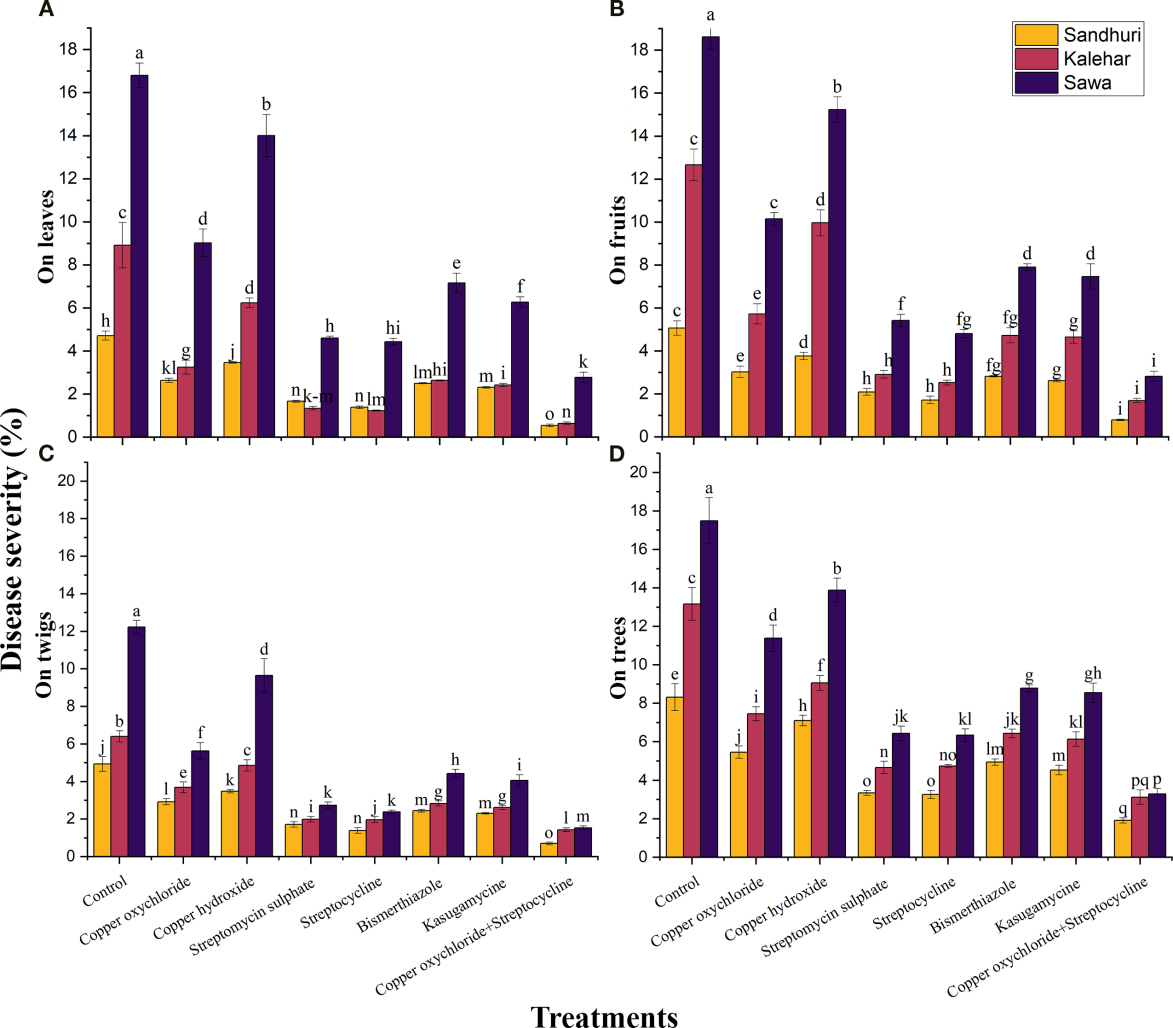
Effect of various antibacterial compounds against bacterial blight severity (%) on Leaves **(A)**, Fruits **(B)**, Twigs **(C)**, and Tree **(D)** on three cultivars of pomegranate. The vertical bars indicate ± standard error (S.E) of means. Means followed by similar letters in each column are not statistically different at *P ≤ 0.05*. LSD value for disease incidence on leaves= 2.55, disease incidence on fruits= 2.82, disease incidence on stem= 2.05, and disease incidence on tree= 0.34.

The interactive effect of bactericides and cultivars on disease severity on leaves, fruits, twigs, and trees was significant (P ≤ 0.05). The lowest disease severity on leaves (0.56%), fruits (0.79%), twigs (0.70%), and trees (1.92%) was recorded in the Sindhuri cultivar when T7 was applied. The same treatment reduced disease severity by approximately 06.7-fold on leaves, fruits, twigs, and trees compared to the control, regardless of cultivar response. The disease severity on leaves, fruits, twigs, and trees on the Sindhuri cultivar was approximately 2.15 and 3.38 times reduced than that of Kalehar and Sava, respectively, irrespective of the treatment effect (**Figure 3**).

#### Disease reduction percentage

**Figure 4:**
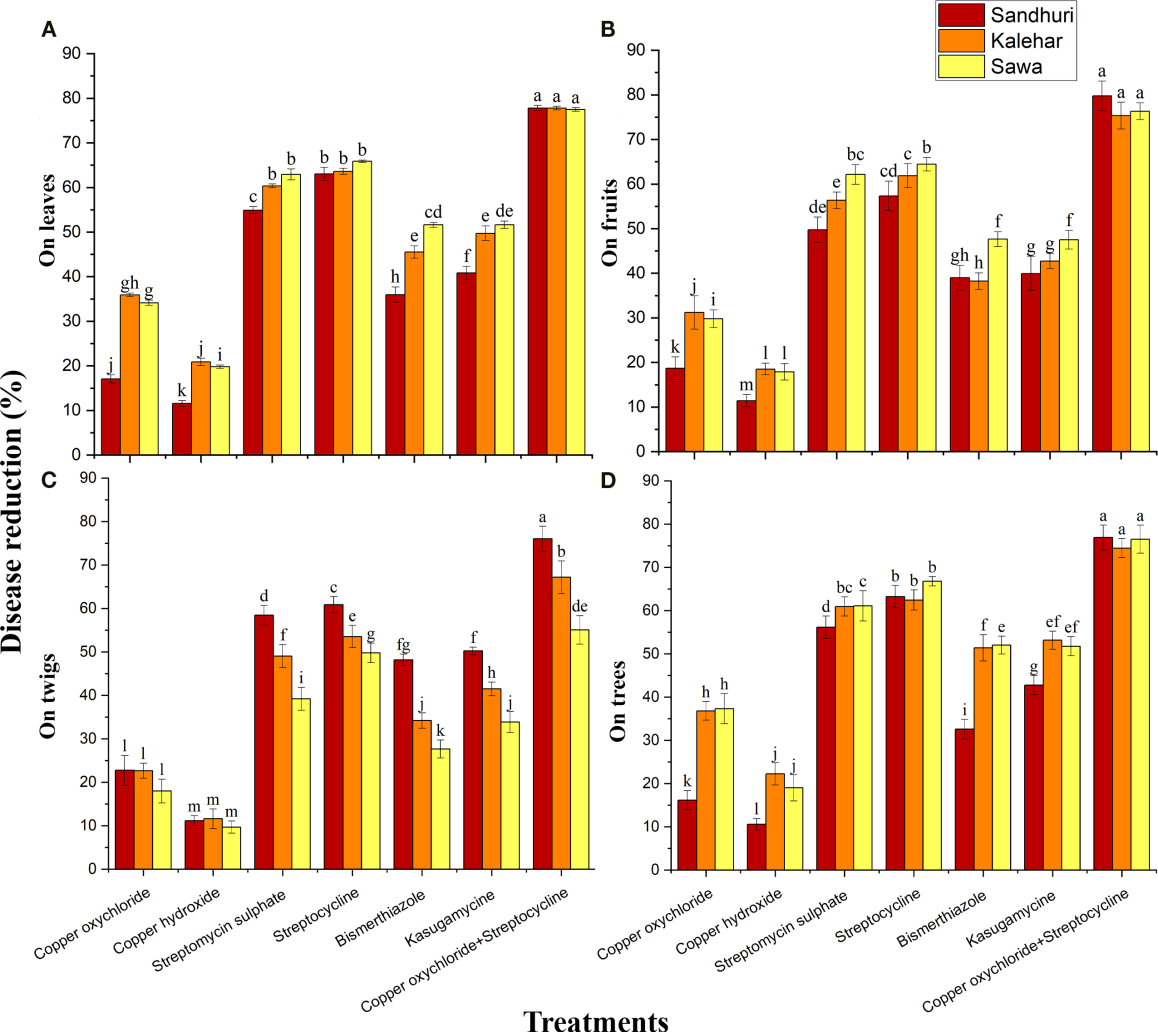
Effect of various antibacterial compounds against bacterial blight disease reduction (%) on Leaves **(A)**, Fruits **(B)**, Twigs **(C)**, and Tree **(D)** on three cultivars of pomegranate. The vertical bars indicate ± standard error (S.E) of means. Bars showing different letters are significantly different at *P ≤ 0.05*.

The interactive effect of bactericides and cultivars on disease reduction percentages on leaves, fruits, twigs, and trees was significant (*P ≤ 0.05*). Maximum disease reduction percentages on leaves (77.63%), fruits (79.76%), twigs (76.1%), and trees (76.9%) were recorded in the Sindhuri when T7 was applied, which was 4.2, 4.1, 6.8, and 4.3-fold higher that of the control on leaves, fruits, twigs, and trees respectively when compared with less effective bactericides (copper hydroxide) regardless of cultivar response. The percentage of disease reduction on leaves, fruits, twigs, and trees in the Sava cultivar was approximately 01.13-fold higher than that in the Kalehar and Sindhuri cultivars (**Figure 4**).

### Physical and biochemical quality attributes

#### Marketable yield, fruit weight, and hundred arils weight

The interactive effect of bactericides on marketable yield, fruit weight, and hundred arils weight (g) was significant (*P ≤ 0.05*). The maximum marketable yield (89.63%) was obtained from the Sindhuri cultivar with T7. The marketable yield received from the same treatment was 1.47 times higher than that of the control, regardless of the cultivar effect. The marketable yield from the Sindhuri cultivar was 1.1 and 1.25-fold higher than those from Kalehar and Sava, respectively. The highest fruit weight (233.1g) and hundred arils weight (42.36g) were found from the Sava cultivar when T7 was applied, 1.63 and 1.89 times more elevated than the control, respectively, irrespective of the cultivar effect. The fruit weight and hundred arils weight (g) obtained from the Sava cultivar were 1.15 and 1.04 times higher than those of the Sindhuri and Kalehar cultivars, respectively, regardless of the treatment effect.

#### Total soluble salts, titratable acidity, and juice pH

The interactive effects of bactericides and pomegranate cultivars related to total soluble salts and juice pH were found to be significant (*P* ***≤*** *0.05*), whereas titratable acidity showed a non-significant interaction. Among the various treatment effects, the highest value of TSS (17.3 °Brix) was obtained from T7, which was 01.39-fold higher than the control, regardless of the cultivar effect. The maximum value of TSS was found by the Sava cultivar irrespective of the treatment effect, which was 1.05 and 1.02 times higher than Sindhuri and Kalehar, respectively. The highest titratable acidity value (0.72%) was obtained from the control, which was 1.28-fold higher when compared to T7, regardless of the cultivar effect. In contrast, the highest value of titratable acidity was found in the Sindhuri cultivar, irrespective of the treatment effect, which was 1.06 and 1.15-fold higher than that of the Kalehar and Sava cultivars, respectively. The highest pH value (4.14) was obtained with T7, which was 1.06-fold higher than that of the control, regardless of the cultivar effect. In contrast, the highest pH value (4.16) obtained from the Sava cultivar, irrespective of the treatment effect, was 1.04 and 1.02-fold higher than those of Sindhuri and Kalehar, respectively (**Table 2**).

**Table 2:** Effect of various antibacterial compounds (Mean ± *S.E) against physical and biochemical parameters of *Punica granatum*.

Cultivar	Treatments (Trade name)	Marketable yield (%)	Fruit weight (g)	Hundred arils weight (g)	Total soluble solids (°Brix)	Titratable acidity (%)	Juice pH
Sindhuri	Control	61.19 ± 0.39*n	137.50 ± 2.64n	19.79 ± 0.21o	11.95 ± 0.05o	0.73 ± .0.003a	3.71 ± .0.004p
	Cobox	69.13 ± 0.61j	165.01 ± 1.39j	24.46 ± 0.13m	13.24 ± 0.20l	0.68 ± .0.001b-d	3.77 ± .0.007n
	Kocide	64.63 ± 0.46l	151.83 ± 1.99m	21.15 ± 0.62n	12.52 ± 0.05m	0.70 ± .0.003b	3.75 ± .0.001o
	Streptomycin	81.94 ± 0.53c	202.43 ± 1.65e	31.20 ± 0.14h	15.40 ± 0.14g	0.63 ± .0.001fg	3.88 ± .0.003i
	Streptocycline	84.94 ± 0.31b	212.28 ± 3.72d	32.67 ± 0.13g	15.70 ± 0.06f	0.62 ± .0.003gh	3.90 ± .0.005gh
	Fornax	74.75 ± 0.25g	177.19 ± 2.07hi	27.68 ± 0.25kl	14.18 ± 0.07j	0.67 ± .0.001de	3.81 ± .0.004m
	Kasomin	77.69 ± 0.45e	187.88 ± 1.06g	29.39 ± 0.17ij	14.68 ± 0.05i	0.65 ± .0.003e	3.84 ± .0.005kl
	Cobox+ Streptocycline	89.63 ± 0.16a	232.74 ± 2.40b	35.43 ± 0.15e	16.23 ± 0.07cd	0.59 ± .0.002ij	3.94 ± .0.004e
Kalehar	Control	54.44 ± 0.71q	148.51 ± 1.09m	21.21 ± 0.37n	12.21 ± 0.09n	0.69 ± .0.002bc	3.83 ± .0.002l
	Cobox	64.72 ± 0.32l	173.00 ± 1.58i	26.82 ± 0.10l	13.75 ± 0.05k	0.65 ± .0.003ef	3.87 ± .0.01j
	Kocide	58.16 ± 0.44o	157.31 ± 0.70kl	23.89 ± 0.93m	13.08 ± 0.04l	0.68 ± .0.001cd	3.85 ± .0.003k
	Streptomycin	73.13 ± 0.28h	212.18 ± 1.07d	35.67 ± 0.16de	15.83 ± 0.09ef	0.59 ± .0.002ij	3.94 ± .0.004e
	Streptocycline	76.31 ± 0.22f	226.43 ± 1.68c	37.04 ± 0.27c	16.23 ± 0.06d	0.58 ± .0.002jk	3.97 ± .0.001d
	Fornax	67.81 ± 0.18k	187.11 ± 0.77g	30.01 ± 0.18i	14.34 ± 0.03j	0.63 ± .0.001gh	3.89 ± .0.003hi
	Kasomin	69.38 ± 0.46j	204.59 ± 0.55e	33.40 ± 0.42fg	14.75 ± 0.07hi	0.61 ± .0.002hi	3.91 ± .0.002f
	Cobox+ Streptocycline	79.72 ± 0.50d	237.03 ± 1.40b	39.30 ± 0.26	16.91 ± 0.15b	0.55 ± .0.003l	3.99 ± .0.005c
Sava	Control	48.69 ± 0.80rs	152.85 ± 1.85lm	21.70 ± 0.60n	12.42 ± 0.13mn	0.65 ± .0.004ef	3.83 ± .0.006i
	Cobox	56.38 ± 0.14p	180.88 ± 1.50h	28.51 ± 0.34jk	14.29 ± 0.04j	0.56 ± .0.001kl	3.90 ± .0.002fg
	Kocide	50.53 ± 0.37r	161.78 ± 0.70jk	24.04 ± 0.57m	13.07 ± 0.05l	0.58 ± .0.003j	3.87 ± .0.00j
	Streptomycin	64.88 ± 0.32l	222.08 ± .90c	36.65 ± 0.18cd	16.08 ± 0.01de	0.50 ± .0.002no	3.98 ± .0.003c
	Streptocycline	69.00 ± 0.43j	235.99 ± 1.51b	39.09 ± 0.25b	16.51 ± 0.02c	0.48 ± .0.003op	4.06 ± .0.003b
	Fornax	61.25 ± 0.24mn	196.13 ± 0.99f	31.34 ± 0.19h	14.94 ± 0.10h	0.54 ± .0.001lm	3.94 ± .0.003e
	Kasomin	62.38 ± 0.52m	211.97 ± 1.88d	33.89 ± 0.31f	15.30 ± 0.08g	0.52 ± .0.002mn	3.97 ± .0.003d
	Cobox+ Streptocycline	71.25 ± 0.24i	245.38 ± 1.76a	43.44 ± 0.39a	17.30 ± 0.14a	0.46 ± .0.003p	4.16 ± .0.003a
	*LSD	1.19	5.65	1.94	0.78	0.02	0.13

*LSD, Least significant difference; *S.E., Standard error; Means followed by the same letters in each column are not statistically different at *p = 0.05*.

### Superoxide dismutase, catalase, and peroxidase

The impact of bactericides on total phenolic contents was significant, but it was non-significant in the case of vitamin C and total antioxidants (AO). The highest value of total phenolic contents (445.46 mg/100g of GAE) was obtained from T7, which was 1.11-fold higher than that of T0, regardless of the cultivar effect. Irrespective of the treatment effect, the total phenolic contents from the Sava cultivar were 1.16 and 1.29 times higher than Sindhuri and Kalehar, respectively. The impact of treatment on SOD was found significant (*P* ***≤*** *0.05*), whereas catalase (CAT) and peroxidase (POD) showed a non-significant (*P* ***≤*** *0.05*) interaction. The highest SOD value (68.30U/mg of protein) was found in the Kalehar cultivar, irrespective of the treatment effect, which was 1.18 and 1.69-fold higher than Sindhuri and Sava cultivars, respectively. The highest value of SOD was obtained from the control, which was 1.28-fold higher than T7 regardless of the cultivar effect. The highest value of POD (1.09 µ/mg of protein) was found in Sava irrespective of the treatment effect, which was 2.15 and 1.88-fold higher than that of the Sindhuri and Kalehar cultivars, respectively. In comparison, the highest value of POD was found in the control, which was 1.24-fold higher than T7, regardless of the cultivar effect (**Table 3**).

**Table 3:** Effect of the exogenous application of bactericides on antioxidant compounds of various pomegranate cultivars. (Mean ± *S.E).

Variety	Treatments (Trade name)	Vitamin C (mg/100g FW)	Total phenolics (mgGAE/100g)	Antioxidant (% inhibition)	SOD* (U/mg of protein)	Catalase (U/mg of protein)	POD* (U/mg of protein)
Sindhuri	Control	23.82 ± 0.43m*	342.03 ± 1.16ij	65.66 ± 0.76c-f	57.62 ± 0.43f	76.56 ± 2.89b	14.28 ± 0.52b
	Cobox	23.99 ± 0.13m	352.37 ± 2.05h	63.32 ± 0.38e-h	53.28 ± 0.61h	73.08 ± 1.11c	12.58 ± 0.21cd
	Kocide	25.50 ± 0.47k	350.72 ± 2.16h	61.30 ± 0.33g-j	56.20 ± 0.07g	66.62 ± 1.94cd	11.74 ± 0.23de
	Streptomycin	26.74 ± 0.15j	367.70 ± 1.72f	57.49 ± 0.17klm	49.07 ± 0.27j	66.00 ± 0.65cd	10.95 ± 0.22ef
	Streptocycline	25.14 ± 0.12kl	369.28 ± 1.99f	58.20 ± 0.21i-m	47.82 ± 0.29k	64.87 ± 1.41d	10.46 ± 0.20fg
	Fornax	24.48 ± 0.15lm	364.00 ± 1.35fg	56.83 ± 1.39lm	50.27 ± 0.16i	68.12 ± 1.18c	11.23 ± 0.16ef
	Kasomin	24.52 ± 0.16lm	361.21 ± 1.99g	62.45 ± 1.48fgh	51.20 ± 0.47i	66.19 ± 1.94cd	11.63 ± 0.09de
	Cobox+ Streptocycline	26.59 ± 0.32j	380.80 ± 1.19e	56.44 ± 0.39lm	43.92 ± 0.39l	60.78 ± 1.25e	09.86 ± 0.23gh
Kalehar	Control	30.18 ± 0.43i	310.89 ± 1.32n	75.39 ± 0.53a	68.37 ± 0.43a	65.75 ± 2.42d	15.44 ± 0.60a
	Cobox	30.46 ± 0.12hi	320.71 ± .99l	69.21 ± 0.57bc	62.83 ± 0.61c	61.03 ± 0.73e	14.26 ± 0.18b
	Kocide	31.90 ± 0.46f	314.50 ± 90mn	67.00 ± 0.21b-e	65.75 ± 0.07b	58.96 ± 0.29f	13.32 ± 0.20bc
	Streptomycin	32.96 ± 0.03e	328.81 ± 1.14k	63.31 ± 0.16e-h	57.62 ± 0.27f	54.28 ± 0.93g	11.82 ± 0.23de
	Streptocycline	31.72 ± 0.11fg	337.04 ± .44j	64.54 ± 0.41d-g	56.37 ± 0.29g	52.47 ± 0.84gh	10.87 ± 0.21ef
	Fornax	30.96 ± 0.15g-i	317.17 ± .82lm	60.67 ± 1.86h-k	60.75 ± 0.47d	49.37 ± 0.67h	11.34 ± 0.17ef
	Kasomin	31.02 ± 0.16gh	317.99 ± 3.35lm	67.49 ± 2.31bcd	59.33 ± 0.38e	50.53 ± 0.59h	11.63 ± 0.15de
	Cobox+ Streptocycline	33.48 ± 0.21de	347.68 ± 2.46hi	54.47 ± 2.85m	53.47 ± 0.39h	49.30 ± 0.96h	10.53 ± 0.24fg
Sava	Control	33.09 ± 0.36e	424.34 ± 1.46d	69.79 ± 0.52b	39.81 ± 0.28m	84.88 ± 2.34a	12.86 ± 0.35c
	Cobox	33.43 ± 0.12de	430.00 ± 1.78cd	63.53 ± 0.54e-h	37.15 ± 0.31o	76.04 ± 1.91b	11.42 ± 0.38ef
	Kocide	34.57 ± 0.26bc	427.98 ± 1.73cd	61.42 ± 0.25ghi	38.63 ± 0.03n	73.18 ± 2.29c	10.53 ± 0.43fg
	Streptomycin	35.18 ± 0.03b	446.84 ± 3.59b	57.67 ± 0.14j-m	34.09 ± 0.23q	66.94 ± 1.67cd	09.38 ± 0.25hi
	Streptocycline	33.95 ± 0.11cd	441.30 ± 1.36b	60.05 ± 0.49h-l	33.42 ± 0.15q	64.54 ± 1.96d	08.42 ± 0.31j
	Fornax	33.68 ± 0.33de	450.85 ± 2.86c	56.02 ± 1.53m	36.02 ± 0.27p	64.05 ± 2.65d	09.49 ± 0.29hi
	Kasomin	34.02 ± 0.16cd	427.14 ± .56cd	62.24 ± 1.98fgh	35.47 ± 0.18p	66.75 ± 0.83cd	09.12 ± 0.19hij
	Cobox+ Streptocycline	36.55 ± 0.22a	465.46 ± 2.02a	57.71 ± 1.09i-m	32.06 ± 0.20r	66.40 ± 1.64cd	08.73 ± 0.41ij
	*LSD	2.01	7.07	5.72	2.75	4.47	0.42

*LSD, Least significant difference; *S.E., Standard error; Means followed by the same letters in each column are not statistically different at p = 0.05, SOD, Super oxide dismutase; POD, Peroxidase.

### Correlation analysis among various antibacterial treatments

**Figure 5:**
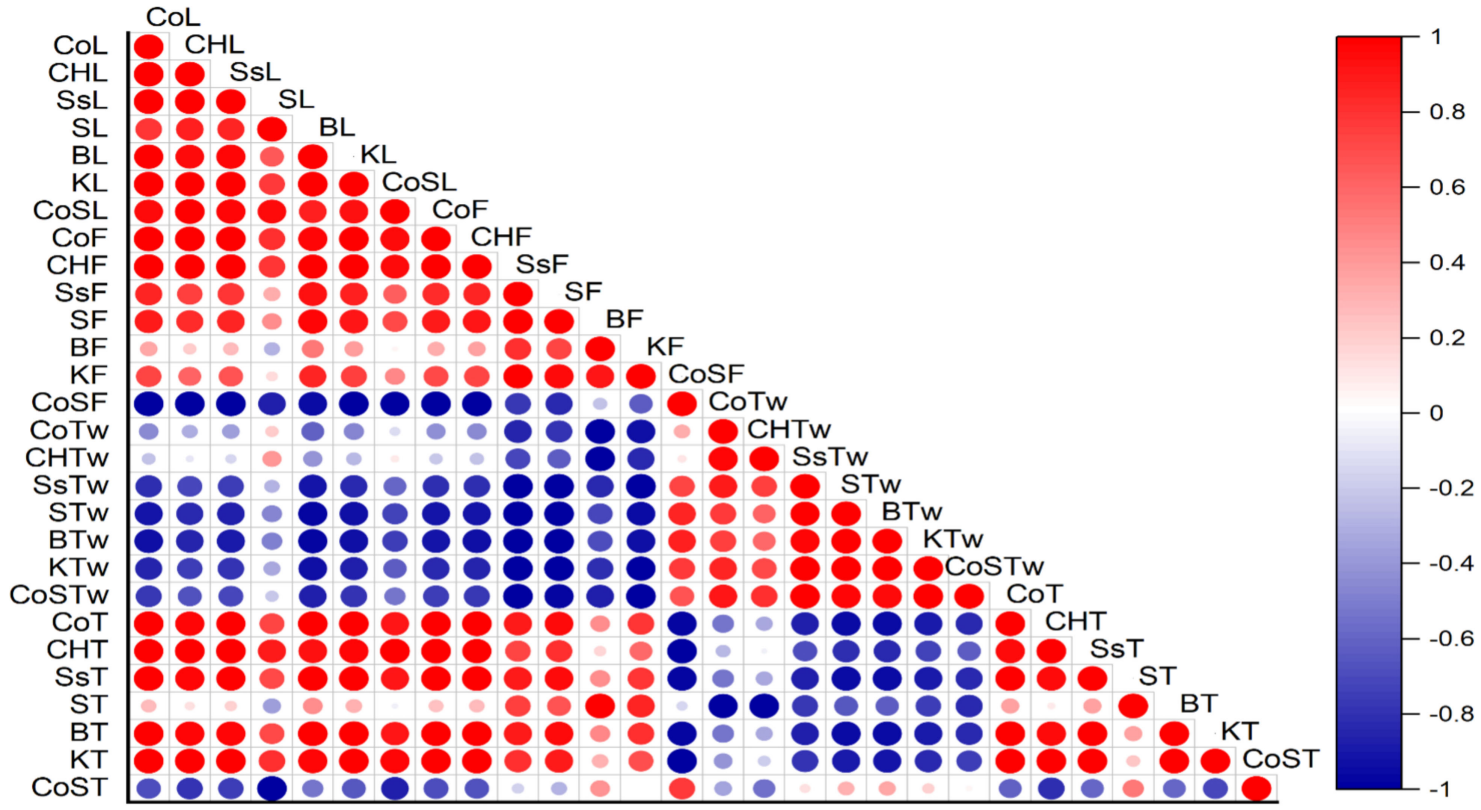
Correlation analysis describes the effectiveness of various antibacterial treatments on disease incidence, severity, and fruit quality attributes in pomegranate cultivars. The abbreviations show the treatments, whereas L, F, Tw, and T represent Leaves, Fruits, Twigs, and Trees, respectively.

The correlation analysis among various antibacterial treatments, including copper oxychloride and streptocycline, and their application as well as the incidence and severity of bacterial blight in pomegranate, and their impact on fruit quality attributes. Each panel (A, B, C, and D) illustrates the correlation between disease incidence (on leaves, fruits, twigs, and trees) and disease severity, as well as the application of different treatments across the three pomegranate cultivars (Sindhuri, Kalehar, and Sava). Positive correlations indicate treatments that effectively reduce disease symptoms, whereas negative correlations indicate minimal impact or ineffectiveness. This analysis describes the potential of certain treatments to enhance the fruit’s biochemical properties, such as TSS, vitamin C, and antioxidant content, as well as to reduce disease severity (**Figure 5**).

### Principal component analysis

**Figure 6:**
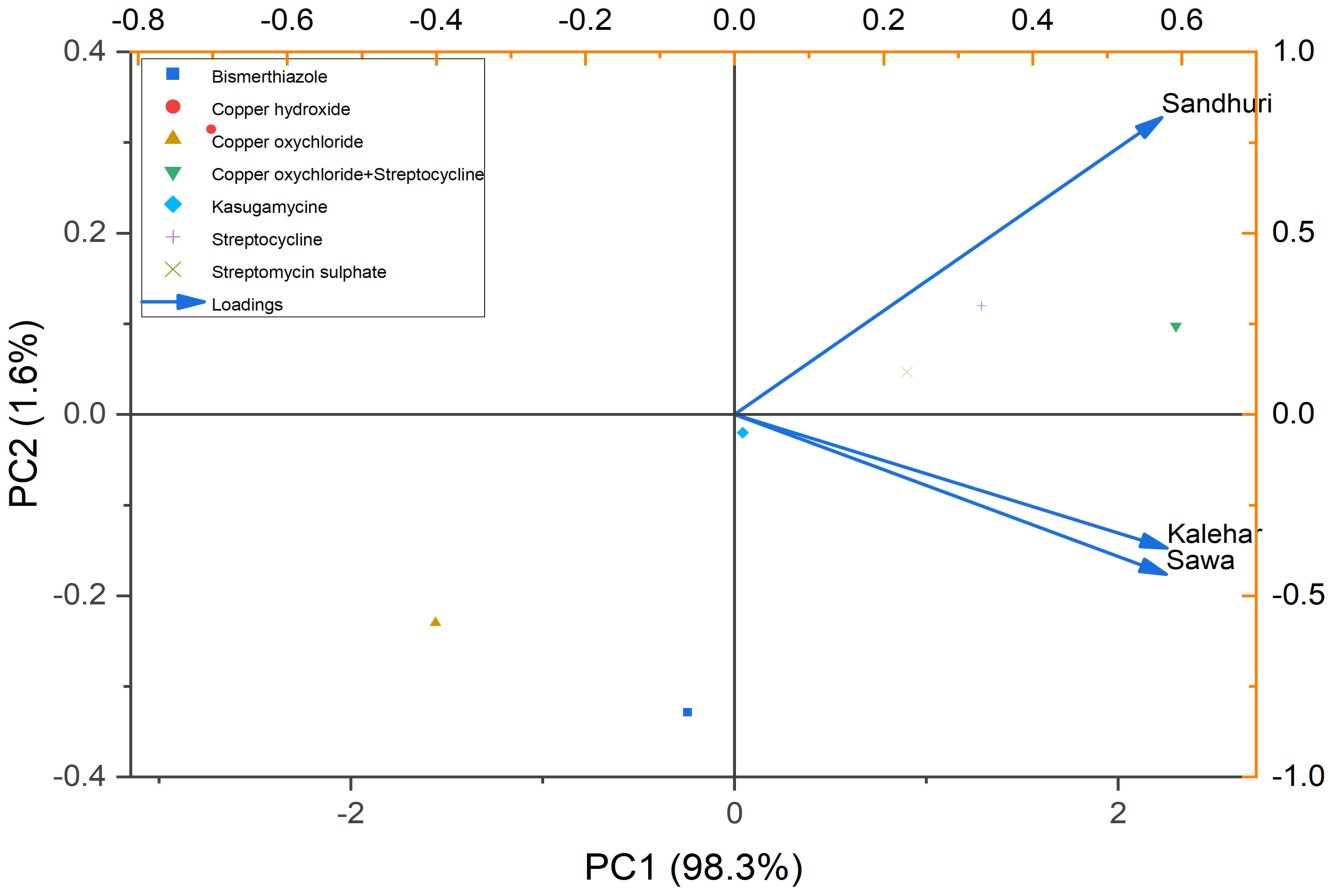
PCA shows the relationship among the pomegranate cultivars (Sindhuri, Kalehar, Sava) and the various antibacterial treatments (e.g., Copper Oxychloride, Streptomycin, Bismuthiazole). The plot highlights the variation in disease reduction across treatments, with PC1 (98.3%) capturing the primary variation. Sindhuri cultivar was found distinct in its response to treatments, while Kalehar and Sava exhibited similar patterns. The vectors represented the loadings of each treatment and cultivar on the principal components, illustrating their influence on bacterial blight control.

The Principal Component Analysis (PCA) biplot describes the relationship between pomegranate cultivars, i.e., Sindhuri, Kalehar, and Sava, and among the various antibacterial treatments, for example, Copper Oxychloride, Streptomycin, and Bismerthiazole. The PC1 axis (98.3%) reported the largest portion of the variance in the data, while the PC2 axis (1.6%) captured less variation. The points on the plot represent the different treatments applied to the plants, while the vectors (arrows) indicate how each cultivar was affected by the treatments. The plot reveals that Sindhuri shows a distinct response compared to the Kalehar and Sava cultivars, particularly in terms of disease reduction or disease severity. Sindhuri was positioned along the positive side of the PC1 axis, highlighting its unique response to the antibacterial treatments. In contrast, Kalehar and Sava were positioned closer to each other in the plot, implying that these two cultivars exhibited similar patterns in response to the treatments applied. This analysis provides valuable insights into how different cultivars react to various bactericides, with Sindhuri showing significant resistance to bacterial blight, which is likely reflected in its position on the PCA biplot. The biplot describes the importance of cultivar selection for managing bacterial blight and optimizing treatment strategies (**Figure 6**).

## Discussion

Bacterial blight of pomegranates is a major disease in various pomegranate-producing regions, causing great qualitative and quantitative losses (Icoz et al., 2014). Removal of the primary source of inoculum, adoption of sanitation practices, and pasting and spraying pruned branches and plant canopies with effective bactericides can help control this disease. Removing the primary source of infection, followed by one spray of copper oxychloride and four sprays of streptocycline (500 ppm) + copper oxychloride (3000 ppm), has been reported to be efficient in reducing the incidence of bacterial canker in acid lime caused by (*Xap*) (Gopal et al., 2004).

The efficacy of treatment T7 in minimizing disease incidence and severity while maximizing disease reduction aligns with established synergistic effects of copper-streptocycline combinations (Raju et al., 2015; Naz et al., 2018). Notably, this formulation’s enhanced performance over individual applications (Lokesh et al., 2014; Yu et al., 2016) stems from complementary bactericidal mechanisms. Copper compounds (e.g., hydroxide/oxychlorides) release cuprous ions (Cu^2+^) that disrupt cell walls and proteins, inhibiting biofilm formation (Mondal and Mani, 2012; Munhuweyi et al., 2016). Concurrently, streptocycline’s aminoglycoside components (e.g., streptomycin) bind the 30S ribosomal S12 protein, terminating protein synthesis (Naz et al., 2017).

The minimum bacterial leaf spot incidence on the grapevine was recorded in plots sprayed with streptocycline (500 ppm) or streptomycin sulphate (500 ppm) by (Ravikumar et al., 2002). Some antibiotics kill and destroy different bacterial populations (Zhang et al., 2014). In the current study, the highest marketable yield was obtained for T7. Our results align with Raju et al (Raju et al., 2015), who recorded marketable yields of 6.12 tons/ha for streptocycline (500 ppm) + copper oxychloride (3000 ppm) and 5.91 tons/ha for streptocycline + copper hydroxide application, and both significantly exceeded control yields. These findings are corroborated by Hussain et al (Hussain et al., 2017, 2019), confirming that streptocycline-copper oxychloride combinations enhance marketable yield percentages in pomegranate.

Our findings corroborate the synergistic potential of chemical-botanical combinations reported by Meena et al. (2017) that *in vitro* screening identified streptocycline (250 ppm) as the most effective antibiotic against the pathogen, followed by tetracycline and neomycin. Among copper compounds, oxychloride (1000 ppm) outperformed hydroxide, while garlic extract (20%) showed superior efficacy among botanicals. Critically, *in planta* trials demonstrated that combined applications, particularly tetracycline (250 ppm) + copper hydroxide (1000 ppm) + garlic extract (20%), achieved significantly greater bacterial blight suppression than individual treatments during consecutive Kharif seasons. This aligns with streptocycline-copper oxychloride combinations in our study, reinforcing how integrated approaches enhance phytopathogen control (Saikia et al., 2004; Maity et al., 2018).

In a study, Tayyab et al. (2023) described four chemicals, such as streptomycin, copper oxychloride, kasugamycin, and oxytetracycline, and four plant extracts, namely *Citrullus colocynthis*, *Calotropis gigantea*, *Acacia nilotica*, and *Moringa oleifera*, which were evaluated against *Xap*. Under laboratory conditions, the results showed that the maximum inhibition zone of bacterial growth was expressed by streptomycin sulfate (38 mm), followed by copper oxychloride (38 mm), kasugamycin (34 mm), and oxytetracycline (24 mm) at 3% concentration after a 72-hour interval. Among the plant extracts, the maximum inhibition was observed in *Moringa oleifera* (27.6 mm), followed by *Acacia nilotica* (26.3mm), *Citrullus colocynthis* (16.6 mm), and *Calotropis gigantea* (19.3 mm) at 25%, 35%, and 45% concentrations (Mubeen et al., 2025). Under laboratory conditions, significant results were obtained for the chemical (copper oxychloride) and plant extracts (*Moringa oleifera*). Copper oxychloride was applied in the greenhouse and showed the lowest disease severity (23%) compared to *Moringa oleifera* (36%) and the positive and negative controls (Manjula et al., 2007).

Moreover, when plants were treated with antibiotics, they showed higher yield, weight, juice (%), peel thickness, peel content, and improved biochemical parameters than non-treated plants (Shokrollah et al., 2011). It was also observed that healthy or asymptomatic fruits had more weight, high juice content, less acidity, and more TSS than bacterially infected fruits (Bassanezi et al., 2009). Maximum fruit weight, juice content (%), TSS, sugar content, and low acidity were achieved with antibiotics treatment in citrus fruits compared to the control (distilled water), which had the highest total acidity, taste, and SOD (Suriachandraselvan et al., 1993; Raju et al., 2015).

However, in another study, ascorbic acid and malic acid contents showed little or no significant difference when antibiotics were applied (Baldwin et al., 2010; Liang et al., 2018). A study reported that bacterial blight of pomegranate, caused by *Xap*, was effectively controlled by applying paushamycin at 500 ppm combined with 0.2% copper oxychloride, administered three times at fortnightly intervals (Baldwin et al., 2010). These findings are consistent with our studies, which also demonstrate that applying bactericides at specific intervals helps reduce disease development and achieve good yields. Karn et al. (2023) found that biochemical analyses revealed a significant increase in all biochemical parameters in pomegranate plants pre-treated with resistance-inducing chemicals (Chen et al., 2022). Foliar application of salicylic acid (SA) and β-aminobutyric acid (BABA) at 300 ppm can protect *Xap*, the causal agent of bacterial blight. They may serve as a potential alternative to chemical pesticides (Farhan et al., 2024). Our results confirm established knowledge that copper-streptocycline combinations effectively suppress *Xanthomonas axonopodis* pv. *punicae* in pomegranate orchards but reveal novel cultivar-specific physiological adaptations. Crucially, Sindhuri exhibited unprecedented metabolic reprogramming under treatment, characterized by enhanced antioxidant pathway activation (CAT: 21.3 U/mg protein; POX: 1.35 U/mg protein) and phytochemical accumulation (TPC: 445 mg GAE/100 mL; vitamin C: 36.5 mg/100 mL). Unexpectedly, Sindhuri’s resilience exceeded all documented benchmarks for South Asian cultivars, reducing disease incidence by >76% across organs while improving marketable yield (89.6 kg/tree). This suggests genotype-dependent optimization of defense pathways during bactericide-induced stress mitigation, a phenomenon previously unreported in pomegranate.

## Conclusion

Bacterial blight is a major constraint to successful pomegranate production. Our study demonstrates that inoculum removal followed by streptocycline-copper oxychloride applications at 15-day intervals (April–August) provides robust efficacy against *Xanthomonas axonopodis* pv. *punicae*, significantly reducing disease intensity while enhancing fruit physicochemical attributes. Critical next steps should prioritize validating this protocol in diverse agroecological zones to establish scalability, assessing long-term impacts on yield stability, soil health, and resistance evolution, and exploring translational applications in other bacterial-pathogen-affected fruit crops (e.g., citrus canker, mango bacterial black spot). This framework offers a sustainable pathway for bacterial disease management in perennial horticulture.

## Data Availability

The original contributions presented in the study are included in the article/supplementary material. Further inquiries can be directed to the corresponding authors.
